# Retina-inspired organic neuromorphic vision sensor with polarity modulation for decoding light information

**DOI:** 10.1038/s41377-023-01310-3

**Published:** 2023-11-07

**Authors:** Ting Jiang, Yiru Wang, Wanxin Huang, Haifeng Ling, Guofeng Tian, Yunfeng Deng, Yanhou Geng, Deyang Ji, Wenping Hu

**Affiliations:** 1 Tianjin Key Laboratory of Molecular Optoelectronic Science, Department of Chemistry, Institute of Molecular Aggregation Science, Tianjin University, 300072 Tianjin, China; 2 Haihe Laboratory of Sustainable Chemical Transformations, 300192 Tianjin, China; 3 State Key Laboratory of Organic Electronics and Information Displays & Institute of Advanced Materials, Nanjing University of Posts & Telecommunications, 210023 Nanjing, China; 4 State Key Laboratory of Chemical Resource Engineering, Beijing University of Chemical Technology, 100029 Beijing, China; 5 School of Materials Science and Engineering, Tianjin University, 300072 Tianjin, China; 6 Tianjin Key Laboratory of Molecular Optoelectronic Sciences, Department of Chemistry, School of Science, Tianjin University, 300072 Tianjin, China; 7 Collaborative Innovation Center of Chemical Science and Engineering, Tianjin University, 300072 Tianjin, China

**Keywords:** Polymers, Photonic devices

## Abstract

The neuromorphic vision sensor (NeuVS), which is based on organic field-effect transistors (OFETs), uses polar functional groups (PFGs) in polymer dielectrics as interfacial units to control charge carriers. However, the mechanism of modulating charge transport on basis of PFGs in devices is unclear. Here, the carboxyl group is introduced into polymer dielectrics in this study, and it can induce the charge transfer process at the semiconductor/dielectric interfaces for effective carrier transport, giving rise to the best device mobility up to 20 cm^2^ V^−1^ s^−1^ at a low operating voltage of −1 V. Furthermore, the polarity modulation effect could further increase the optical figures of merit in NeuVS devices by at least an order of magnitude more than the devices using carboxyl group-free polymer dielectrics. Additionally, devices containing carboxyl groups improved image sensing for light information decoding with 52 grayscale signals and memory capabilities at an incredibly low power consumption of 1.25 fJ/spike. Our findings provide insight into the production of high-performance polymer dielectrics for NeuVS devices.

## Abstract (web summary)

The polymer dielectrics with carboxyl groups are used to investigate the polarity modulation effect, which can effectively modulate a device to simulate artificial vision systems and to decode light information.

## Graphical abstract

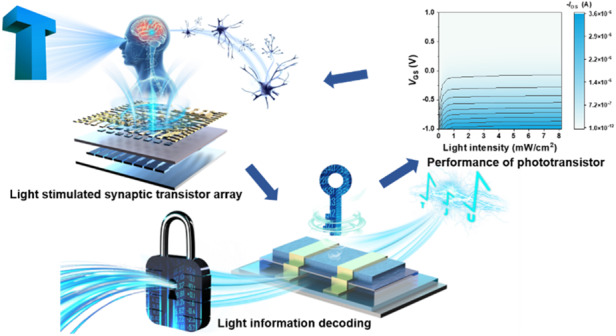

## Introduction

Development of various fields such as electronic skin, photonic synapse, sensor detection, wearable electronics artificial intelligence, etc.^1–7^ are clear evidence of technological advancements in the field of organic electronics. For the performance modulation of organic electronic devices, the interface is commonly present and is considered to be important^8–12^. Considering organic field effect transistors (OFETs) and OFETs-based photonic devices, the interface quality between insulators and semiconductors is of utmost importance for the distribution of effective charge^13–17^. Some insulators specifically polymer dielectrics are highly capable of the construction of superior interfaces due to their supremacy in mechanical flexibility and superficial operability^18–23^. Hence, polar functional groups (PFGs) provide the polymer insulators with the maximum likelihood to affect the distribution of charge^19,24–34^. According to some studies, fluorine-containing PFGs could improve efficacy by lowering or increasing the interfacial trap and charge densities^24^ in the conduction channel due to the effect of the dipole process^25^. However, as far as our knowledge is considered, many researchers have revealed that polar hydroxyl (–OH) containing functional groups in polymer dielectric layers could produce interfacial charge traps to inhibit charge distribution throughout the channel layer that results in the diminution of the activity. Functional groups such as hydroxyl (–OH), ether (–O–), and ester (–COO–) prefer to merge with H_2_O and O_2_ in the settings for the formation of charge traps to lower the performance^19,26–32^. Some of the reports have demonstrated that polar hydroxyl-containing functional groups could improve the device activity^32–34^. As a result, the mechanism of polar hydroxyl-containing functional groups on device performance is not sufficiently clear, and only a few reports are concerned about their effect on photogenerated carriers in NeuVS devices, which is critical for understanding the relationship between the chemical structure of polymer dielectric and properties of a device.

In this paper, we synthesize two types of polymers with similar chemical structures, poly amic acid (PAA) with a carboxyl group (–COOH) and polyamide (PA) devoid of –COOH group, as dielectric layers in low-voltage OFETs, which are combined with Dinaphtho [2,3-b:2’,3’-f] thieno[3,2-b] thiophene (DNTT) as an active layer to test the polarity modulation effect in the performance of a device. As a result, the presence of –COOH aids in the formation of more orderly packed molecular layers in the conducting channel and preferentially induces charge transfer at semiconductor and dielectric interfaces for high-performance OFETs, with the best value reaching 20 cm^2^ V^−1^ s^−1^. Furthermore, the optical figures of merit in PAA-based phototransistors, which include photosensitivity, photoresponsivity, and detectivity, are at least an order of higher magnitude than that in PA-based phototransistors. Additionally, photonic synaptic instruments using PAA as dielectric layers reveal better image sensing and memorizing ability as compared to polyamide-based devices.

## Results

### Simulation calculation and characterization

**Fig. 1:**
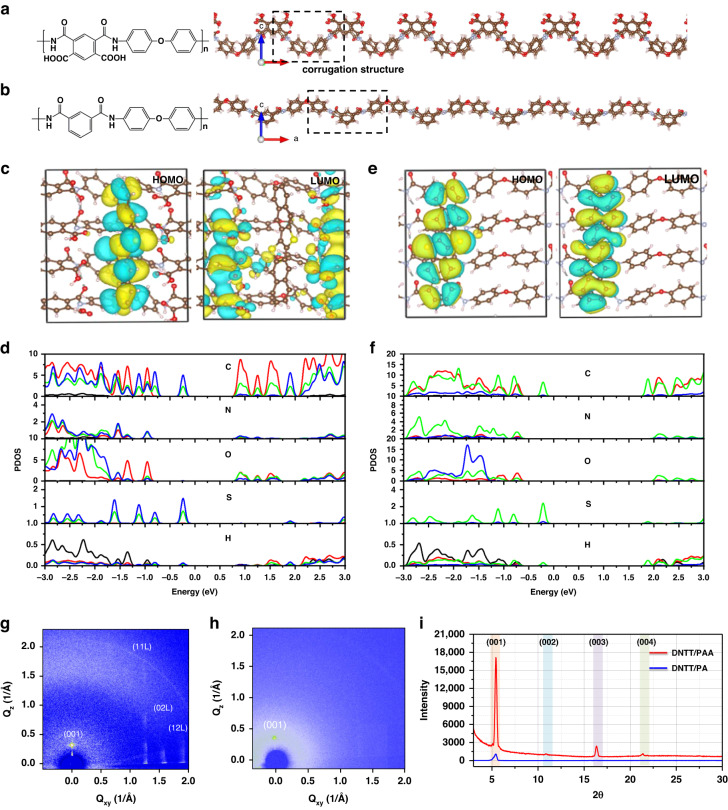
Atomistic simulation, theoretic calculation, and film characterization. The molecular structure and secondary structure of **a** PAA and **b** PA. The calculated HOMO and LUMO of **c** PAA/DNTT system and **e** PA/DNTT system. Partial density of states (PDOS) of **d** PAA/DNTT system and **f** PA/DNTT system. 2D GIXRD patterns of DNTT (20 nm) on the surface of PAA (**g**) and PA (**h**). **i** XRD patterns of DNTT films (20 nm) grown on PAA and PA surface

The molecular structures of PAA and PA are depicted in Fig. 1a, b, whereas the synthetic route to PAA is based on our previous report^33^, and the details of synthesizing PA are described in the “Materials and methods” section. Atomistic simulations were used to investigate the secondary structures of PAA and PA, as shown in Fig. 1a. It was clear that the distance of the corrugation structure in the PA molecule was greater than that in the PAA molecule due to the weak interaction of corrugations without OH groups, which might have an impact on the growth of organic semiconductors. Furthermore, the Gaussian 09 package was used to investigate the basic units of PAA and PA. At the DFT/B3LYP/6-31G (d) level, the optimized geometry at ground state, electrostatic potential (ESP) map, and lowest unoccupied molecular orbital (LUMO) were calculated, indicating that PAA had a stronger electron-withdrawing ability compared to PA (Fig. S1). In addition, density functional theory (DFT) calculations were carried out to better understand the interaction at the interface. Our calculations showed that in the PAA/DNTT system, the highest occupied molecular orbital (HOMO) was primarily distributed on DNTT and the lowest unoccupied molecular orbital (LUMO) was primarily distributed on PAA (Fig. 1c), with an energy band gap of 1.5 eV, indicated that excited electrons could transfer from DNTT to PAA. HOMO was primarily composed of pz and px orbits of C in DNTT, pz and px orbits of S, and py orbits of O in PAA when merged with the partial density of state (PDOS) (Fig. 1d), whereas LUMO was primarily composed of py and pz orbitals of C and O elements in the carboxyl group of PAA. As a result, the small energy band gap of DNTT–PAA and the different spatial distributions of HOMO–LUMO promoted electron excitation, separation, and migration. In comparison, both HOMO and LUMO were distributed on DNTT (Fig. 1e, f) with a 2.4 eV energy band gap in the PA/DNTT system, indicating that no charge transfer occurred at the interface, and charge carriers were difficult to separate and migrated at this interface. Furthermore, our calculations showed that the presence of a –COOH group could enhance the interaction between the dielectric layer and the semiconductor (Fig. S2), resulting in more orderly packing during the growth of molecules.

The active layer DNTT was deposited onto the surface of PAA and PA for further characterization to evaluate the effect of dielectric layers on semiconductor growth. The structural order of the deposited DNTT films on these two types of polymer dielectrics was primarily detected using 2D grazing-incidence X-ray diffraction (GIXRD). The diffraction peak of DNTT thin films on the PAA surface (Fig. 1g) in the direction of Qz (out-of-plane) and Qxy (in-plane) reflections was more powerful than that on the PA surface (Fig. 1h), indicating more orderly molecular stacking and higher crystalline film on the PAA surface for more efficient charge transport. Furthermore, the X-ray diffraction (XRD) patterns shown in Fig. 1i showed only high-ordered and highly crystalline DNTT films forming on the PAA surface display only (00*l*) lattice planes. In comparison, the deposited DNTT films exhibited comparable low order and crystallinity on the PA surface (Fig. 1i). Furthermore, the morphology of DNTT films on these two types of dielectric surfaces was investigated using atomic force microscopy (AFM), indicating that there will be more traps induced by the crystal structure in the conducting channel above the PA dielectric, obstructing effective charge transport. Surface-enhanced Raman scattering was observed from the DNTT/PAA surface, with the intensity of the Raman spectrum being more powerful than that on the PA surface, as shown in Fig. S3, represented a much stronger contact between the PAA and DNTT, in accordance with the theoretical calculation results.

### Electrical and photoresponse characterization of the devices

**Fig. 2:**
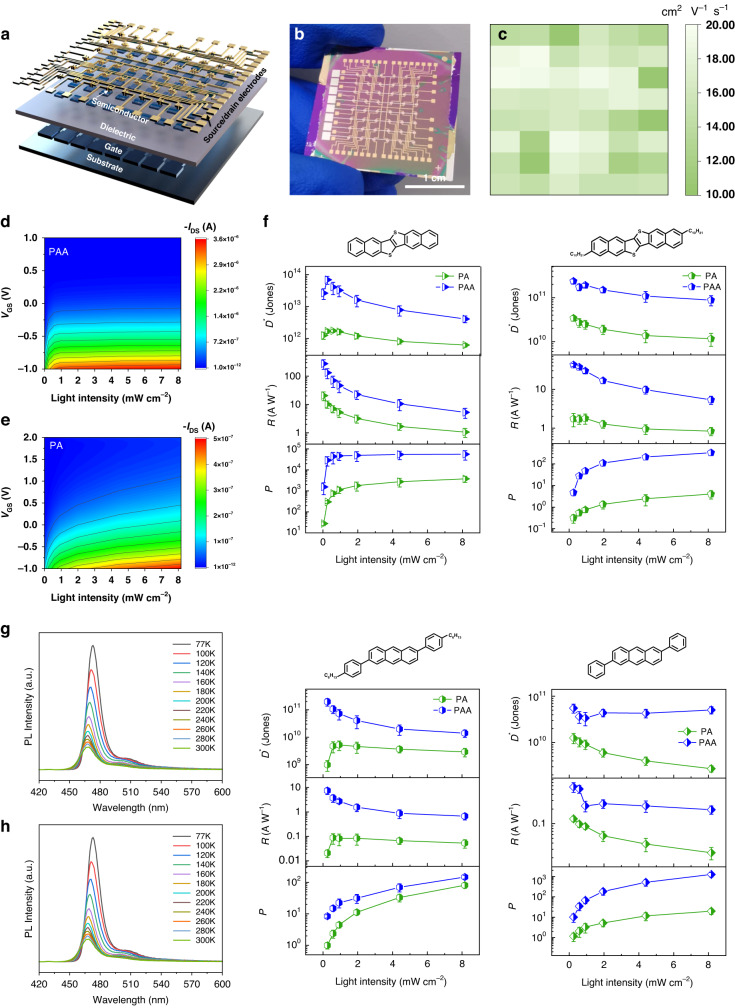
Performance of OFET array and phototransistors based on PAA and PA. **a** Schematic diagram of the device structure of OFET. **b** Physical diagram of device arrays. **c** Distribution of PAA-based OFET arrays mobilities. **d** Photocurrent of PAA-based phototransistor measured under different illumination intensities in the air. **e** Photocurrent of PA-based phototransistors measured under different illumination intensities in the air. **f** *P*, *R*, and D^*^ as a function of illumination intensity using PA and PAA as the dielectric layers with different semiconductors. The values and error bars in **f** were the mean value and standard deviation obtained with 10 OPT devices. Temperature-dependent photoluminescence (PL) spectra for DNTT/PAA (**g**) and DNTT/PA films (**h**) excited at 400 nm

OFET arrays with bottom-gate top-contact (BGTC) setup, PAA and PA as insulator layers (Fig. 2a, b), were built to explore the polarity attenuation influence on low-voltage organic transistors. For PAA-based devices with carboxyl groups (–COOH), all the devices have mobility values >10 cm^2^ V^−1^ s^−1^, with the best value reaching 20 cm^2^ V^−1^ s^−1^ (Fig. 2c) from the saturation mode at a low operation voltage of −1 V and an ON/OFF ratio of 10^5^–10^6^ (Fig. S4 and Table S1). The devices using the PAA dielectric exhibited gate modulation in both the linear and saturation regimes. In contrast, devices using the PA dielectric layer without the carboxyl group (–COOH) not only achieved mobility of around 0.5 cm^2^ V^−1^ s^−1^ but also showed a low output of the devices (Fig. S5). Additionally, from the linear area of the output curve, it could be observed that the comparatively maximum contact resistance was found in the PA device. Meanwhile, as seen between the forward and backward sweeps in Fig. S6, carboxyl groups can reduce the hysteresis effect of PAA-based devices in the dark due to a small interface trap. Moreover, the calculated activation energy (*E*_a_) could also validate a low grade of energetic condition^35^ at the interface of PAA/DNTT (*E*_a_ = 27.5 meV) comparatively with the interface between the PA and DNTT (*E*_a_ = 70.2 meV). Based on this, it could be established that based on charge carrying capacity PAA/DNTT interface could be transferred more significantly. It was also indicated the efficacy of the carboxyl polarity group influenced the operational charge transport. Other three semiconductors, such as 2,6-diphenylanthracene (DPA), 2,6-bis(4-hexylphenyl) anthracene (C6-DPA), and 2,9-Didecyldinaphtho[2,3-b:2’,3’-f]thieno[3,2-b]thiophene (C10-DNTT), were employed to construct the OFETs, and the performance was listed in Table S2. In addition, three types of polymer dielectrics (polystyrene (PS), poly (methyl methacrylate) (PMMA) and poly (propylene oxide) (PPO)) without –COOH were chosen as buffer layers to decrease the impacts of the carboxyl group, and it was discovered that mobility significantly reduces and operating voltage increases. In comparison, devices with a single component dielectric layer were investigated (Fig. S7), and their performance was lower than those of the PAA/buffer layer system. Both approaches showed that even from the bottom layer, the carboxyl group could efficiently influence charge transfer in the conducting channel.

Following that, the effect of the carboxyl group on photogenerated carriers based on organic phototransistors (OPTs) was investigated further. To irradiate the devices, monochromatic light at 450 nm with varying light density was used. The output photocurrent rose with increasing light density until it reached saturation at high density, and it was found that the device with PAA as the dielectric layer was more responsive to illumination (Fig. 2d) than that with PA as the insulating layer (Figs. 2e and S8). As a result, optical figures of merit such as photosensitivity (*P*), photoresponsivity (*R*), and detectivity (D^*^) were improved by at least one order of magnitude with effective performance in PAA-based devices when they were compared to PA-based phototransistors (Fig. 2f). Temperature-dependent photoluminescence (PL) spectra tests were also performed to better understand the exciton dissociation kinetics in these two types of DNTT/PAA (Fig. 2g) and DNTT/PA films (Fig. 2h). Both films exhibited the phenomenon of red-shifted PL peaks and increased PL intensity as temperature decreases. As a result, the energy barrier (*E*_*b*_) was low value for DNTT/PAA films (31.9 meV) and high value for DNTT/PA films (34.1 meV) based on Arrhenius equation^36^ (Fig. S9), which provides favorable evidence for the positive effect of carboxyl group on photogenerated carriers. Additionally, phototransistors based on the other three semiconductors, DPA, C6-DPA, and C10-DNTT, were also produced in order to demonstrate the wide applicability of the carboxyl group’s beneficial effect on photogenerated carriers. The performances (Fig. 2f) confirmed this broad applicability.

### Synaptic behaviors of the devices

**Fig. 3:**
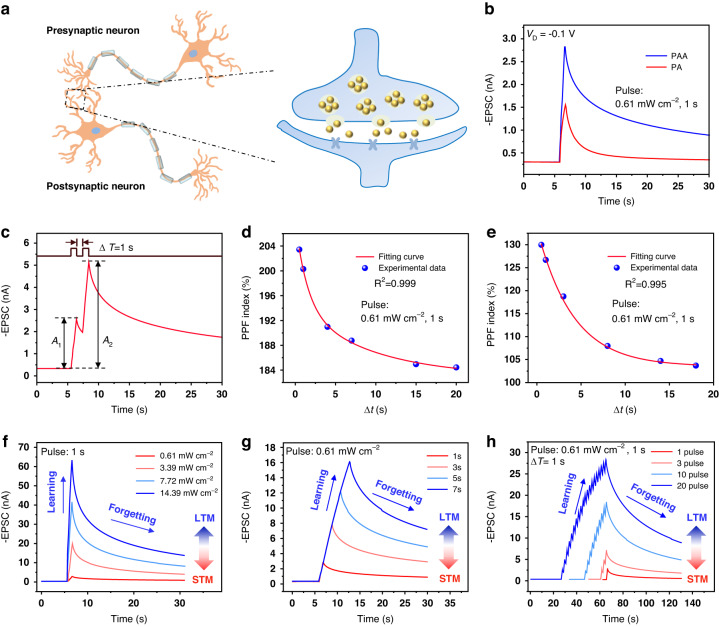
Light-stimulated synaptic characteristics. **a** Schematic diagram of neurotransmitter transmission through synapses between adjacent neurons. **b** EPSC trigged by a light spike (450 nm, 0.61 mW cm^−2^, 1 s) with a constant *V*_D_ of −0.1 V and *V*_G_ of 0.12 V. **c** EPSC trigged by a pair of light pulses (450 nm, 0.61 mW cm^−2^, 1 s) with a constant *V*_D_ of −0.1 V and *V*_G_ of 0.12 V for PAA device. Dependence of the PPF index for **d** PAA device and **e** PA device on Δ*t*. The relationship between EPSC of PAA device and **f** light pulse intensity, **g** light pulse width, and **h** light pulse number (source–drain voltage *V*_D_ = −0.1 V, gate voltage *V*_G_ = 0.12 V)

Moreover, the influence of the carboxyl group in polymer on the light-stimulated synaptic^37^ function of devices was investigated, inspired by biological synapses in the human visual system (Fig. 3a). Figure 3b showed the typical excitatory postsynaptic current (EPSC) of the DNTT synaptic transistors based on PA and PAA dielectric initiated by a light spike (450 nm, 0.61 mW cm^−2^, 1 s) with a constant drain voltage (*V*_D_) of −0.1 V and gate voltage (*V*_G_) of 0.12 V. When a light spike was applied, the EPSC of the PA device reached a peak value of 1.6 nA and dropped significantly to 26% of the initial current after the presynaptic light stimulation was removed for 10 s, whereas the EPSC of the PAA device remains higher than 45% of the initial current (2.8 nA), indicating a greater retention time. Furthermore, paired-pulse facilitation (PPF) is a type of short-term memory (STM) that referred to the concept in which the current caused by the second time (*A*_2_) was stronger than the current caused by the first time (*A*_1_) after two successive presynaptic stimuli (Fig. 3c), and it played an important role in real-time decoding/recognition of visual or auditory information for the biological nervous system^38^.

The PPF index of the PAA-based device gradually reduces as the Δ*t* increases from 0.5 to 20 s as represented in Fig. 3d. The reduction trend could be fitted well by the double exponential function^39^

$${{\rm {PPF}}}={C}_{0}+{C}_{1}\times \exp \left(-\frac{\triangle t}{{\tau }_{1}}\right)+{C}_{2}\times \exp \left(-\frac{\triangle t}{{\tau}_{2}}\right)$$

where *τ*_1_ and *τ*_2_ refer to the characteristic relaxation times of the rapid and slow phases, respectively. For biological synapses, *τ*_2_ is about one order of magnitude greater than *τ*_1_, and a high PPF index in optoelectronic synaptic devices was crucial to promoting the development of artificial vision. Here, *τ*_1_ and *τ*_2_ from the fitting results based on PAA devices were found to be 1.47 and 11.32 s, respectively, in which the maximum PPF (*A*_2_/*A*_1_) index of the PAA devices exceeded 200%. For the sake of comparison, the maximum PPF index of the PA devices was only 130% (Fig. 3e). For further comparing the simulation effect of PAA and PA devices on biological synaptic behavior, we examined the synaptic plasticity of the two devices. For the brain, memory behavior could be categorized into short-term memory (STM) and long-term memory (LTM) based on retention time. Long-term memory could be formed through successive training and augmentation based on short-term memory. The transition from STM to LTM is shown in Fig. 3f–h. The transformation was successfully obtained by the alteration of the intensity of the light pulse, by varying the light pulse width and changing the number of light pulses of PAA devices. In the case of PA devices (Fig. S10), when light stimulation was forcefully applied, its EPSC would tend to saturate at a much faster rate, and the retention time when compared with the application of a single light pulse, did not broaden remarkably, definitely showing sub-standard synaptic plasticity than PAA devices. It was noteworthy that using illumination of 0.61 mW cm^−2^ from both PAA and PA-based synaptic OPT devices, a quick time response of 20 ms could be attained. Short-term plasticity (STP) for PA-based devices was detected instead of long-term plasticity (LTP), which suggested that devices using PA as the dielectric would likely exhibit the potential STM characteristic.

**Fig. 4:**
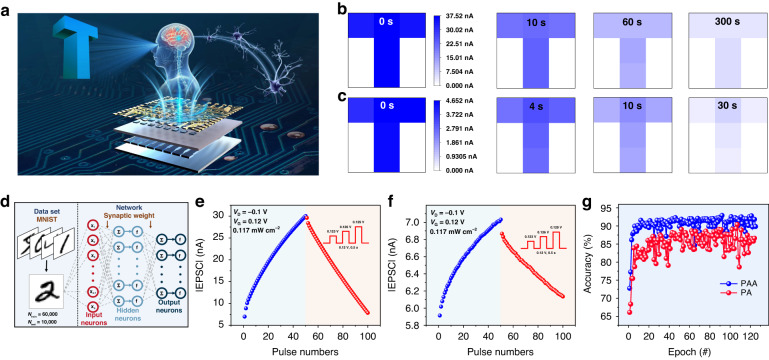
Simulation of image memory and recognition ability. **a** Schematic diagram of a T-shaped light-stimulated synaptic transistor array. Image sensing and memory process of the **b** PAA and **c** PA devices by using a T-shaped light-stimulated synaptic transistor array. **d** Schematic illustration of the multilayer neural network for digit recognition. LTP/LTD characteristics curve for PAA **e** device and PA **f** device. **g** Accuracy rate as a function of training epochs

Additionally, our devices successfully mimicked STM–LTM conversion, the brain’s “learning–forgetting–relearning” process and PAA-based devices show better relearning ability (Fig. S11). On this basis, we showed the image sensing and memory processes of the two devices using T-shaped light-stimulated synaptic transistor arrays (Fig. 4a) stimulated by a light pulse of 0.61 mW cm^−2^ for 10 s with a *V*_G_ bias of 0.12 V. The forgetting process for PAA devices (Fig. 4b) is relatively fast at first, followed by a slow forgetting process, and finally significantly lowered to the initial current level after 5 min, whereas for PA devices (Fig. 4c), the current could be forgotten to the initial level only after 30 s, implying that PAA-based devices have better image recognition and memory capabilities.

Furthermore, the low energy consumption of synaptic devices was critical for simulating human brain learning behavior^40^. Even at ultralow operating voltages, our devices exhibited obvious EPSC behavior. As a result, the calculated energy consumption of PAA and PA devices per synaptic event was 1.25 and 0.703 fJ (Fig. S12) with an operating voltage of −1 mV and a pulse time of 0.1 s, respectively, which was equatable to the biological level (1–10 fJ per synaptic event)^41^ and was very promising in simulating artificial neuromorphic systems. A neural network is a computing model that uses a large number of computing neurons to simulate the human brain. These neurons are connected to each layer via synaptic weight. Each layer of neurons can exhibit parallel computing and transfer information at a large scale between them^42^. As a result, we conducted an artificial neural network (ANN) simulation and calculation based on the Modified National Institute of Standards and Technology (MNIST) database (Fig. 4d)^43^ to further investigate the potential of the two devices in the application of artificial visual perception systems. As shown in Fig. 4e, f, long-term potentiation (LTP) of synaptic weight was obtained by 50 consecutive light pulses (0.117 mW cm^−2^, 0.5 s, without electric pulses) and long-term depression (LTD) synaptic weight was obtained by 50 consecutively increasing electric pulses (without light pulses). The overall identification precision of the two devices experienced a rapid growth stage at the start and gradually saturated as the training period increased, eventually reaching 88% (PA-based devices) and 93% (PAA-based devices) (Fig. 4g), respectively, representing a remarkable application perspective in neuromorphic computing.

### Applications of PAA-based devices for light information decoding

**Fig. 5:**
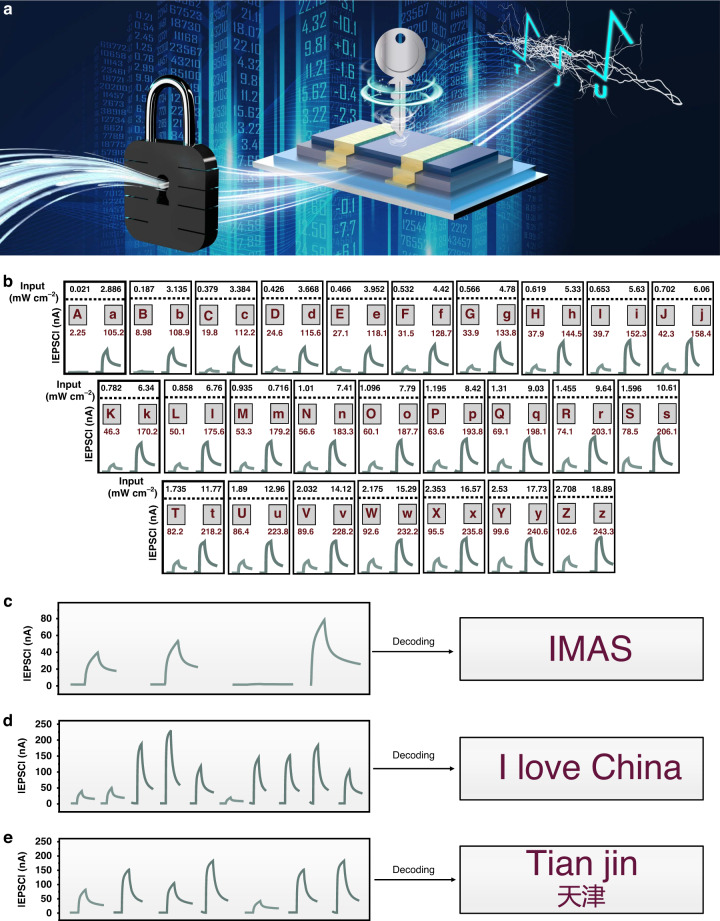
Device performance for light information decoding. **a** Schematic diagram of the light information decoding process. **b** Code book with 52 grayscale signals for Aa to Zz. **c** “IMAS” decoding process. **d** “I love China” decoding process. **e** “Tian jin, ” decoding process

Our phototransistor-based synaptic devices using PAA as dielectrics could also display discriminative electrical signal responses to different light intensities, indicating potential application in light information decoding, in addition to consecutive light pulses with the same light intensity for information recognition (Fig. 5a). It is interesting to note that our manufactured devices demonstrated a clear light response to 52 grayscale signals between 0.021 and 18.99 mW cm^−2^, which exactly matched 52 English characters in both upper and lower case. Based on this, we can create a code book (Fig. 5b) that combines light intensities and photoresponse current signals to decode any encrypted content in English or Chinese pinyin. For instance, the phrase “I love China” and the acronym “IMAS” (Institute of Molecular Aggregation Science, Fig. 5c) are both easy to decode (Fig. 5d). Furthermore, information can be derived from Chinese pinyin that has a unique light information signal, such as Tian jin (Fig. 5e).

## Discussion

In conclusion, the effective role of the carboxyl group in polymer dielectrics for OFETs and OFET-based photonic devices has been demonstrated. Its role not only dominated effective charge distribution but can modulate photo-generated carriers. The theoretical calculation also showed that the transfer of charge that occurred at the semiconductor/dielectric played an important role due to the presence of an electron-withdrawing group (–COOH). As a result, our OFETs could achieve mobility of up to 20 cm^2^ V^−1^ s^−1^ at a −1 V operation voltage. When dielectric materials with polar carboxyl groups were utilized, phototransistors-based photonic synaptic devices also worked well for light information decoding applications. Our findings open up new avenues for the development of high-quality polymer dielectrics with polar functional groups.

## Materials and methods

### Polymerization of polyamide (PA)

Taking the PA with a solid content of 15% as an example, LiCl (2 g), 4,4’-oxybisbenzenamine (ODA, 12.3686 g), and imidazole (4.2050 g) were stirred and dissolved in DMAc solution at room temperature. Then 12.5403 g of isophthaloyl dichloride was added to the solution in batches. For 4–8 h, the solution was continuously stirred in an ice bath and a N_2_ atmosphere. The following is the synthetic route to PA.
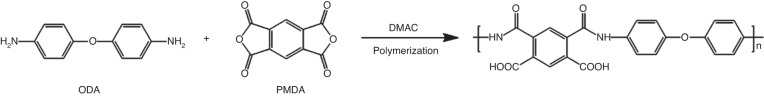

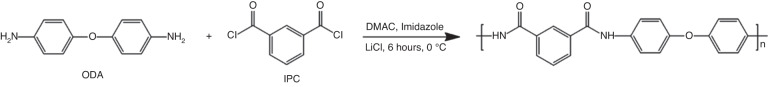


### Materials

DNTT, DPA, C6-DPA, C10-DNTT, PS, PMMA, and PPO were purchased from Sigma-Aldrich. DNTT, DPA, C6-DPA, and C10-DNTT were directly used for vacuum deposition.

### Measurements

The electrical characteristics of the OFET and OFET-based devices were recorded at room temperature in the air by using a Keithley 4200 SCS semiconductor parameter analyzer and a Micromanipulator 6150 probe station. Atomic force microscopy (AFM) measurements were performed using a Multimode Nanoscopy IIIa instrument (Digital Instrument) in tapping mode with silicon cantilevers. The plasma treatment was carried out using Gala Instrument Prep2. XRD measurements were performed using a Rigaku Smartlab diffractometer in reflection mode at 45 kV and 200 mA with monochromatic Cu Ka radiation.

### Sample preparation

Bottom-gate top-contact OFETs were fabricated, the Si/SiO_2_ wafer was only used as the device substrate, and Si and SiO_2_ were not used as the gate and dielectric layer of the device. Firstly, 100 nm of Al gate was vacuum deposited on the substrate with a mask, then the surface of substrate with Al was treated with O_2_ plasma (50 W, 1 min), a flat thin film of PAA (annealing-free) and PA (140 °C annealing for 1 h) was obtained by spin coating PAA (in the air) and PA (in the glovebox) solution (in the DMAc solvent) on top of the substrate with Al. As tested from independent Au/PA (65 nm)/Al structures, Ci of the PA dielectric is determined as 69 nF cm^−2^ at 1 kHz (Fig. S13). The Ci of the PAA dielectric was calculated to be 50 nF cm^−2^, and the semiconductors with 20 nm thickness were vacuum deposited on the polymer dielectrics with a mask. Finally, to form top-contact devices, a 20 nm-thick Au film was deposited on the semiconductor layers as a source–drain electrode with a mask.

### Supplementary information

Supplementary Information for Retina-inspired Organic Neuromorphic Vision Sensor with Polarity Modulation for Decoding Light Information
