# Supplementary material for: Retina-inspired organic neuromorphic vision sensor with polarity modulation for decoding light information
Source: Light Sci Appl. 2023 Nov 7;12:264. doi: 10.1038/s41377-023-01310-3 (PMC10628194; doi:10.1038/s41377-023-01310-3)

**Supplementary Information for:**

**Retina-inspired** **Organic Neuromorphic Vision Sensor with Polarity Modulation for Decoding Light Information**

Ting Jiang^1,2^, Yiru Wang^3^, Wanxin Huang^3^, Haifeng Ling^3^, Guofeng Tian^4^, Yunfeng Deng^5^, Yanhou Geng^5^, Deyang Ji^1,2^* and Wenping Hu^2,6,7^

^1^Tianjin Key Laboratory of Molecular Optoelectronic Science, Department of Chemistry, Institute of Molecular Aggregation Science, Tianjin University, Tianjin 300072, China.

E-mail: [jideyang@tju.edu.cn](mailto:jideyang@tju.edu.cn)

^2^Haihe Laboratory of Sustainable Chemical Transformations, Tianjin 300192, China.

^3^State Key Laboratory of Organic Electronics and Information Displays &Institute of Advanced Materials, Nanjing University of Posts & Telecommunications, Nanjing 210023, China.

^4^State Key Laboratory of Chemical Resource Engineering, Beijing University of Chemical Technology, Beijing 100029, China

^5^School of Materials Science and Engineering, Tianjin University, Tianjin 300072, China.

^6^Tianjin Key Laboratory of Molecular Optoelectronic Sciences, Department of Chemistry, School of Science, Tianjin University, Tianjin 300072, China.

^7^Collaborative Innovation Center of Chemical Science and Engineering, Tianjin University, Tianjin 300072, China.


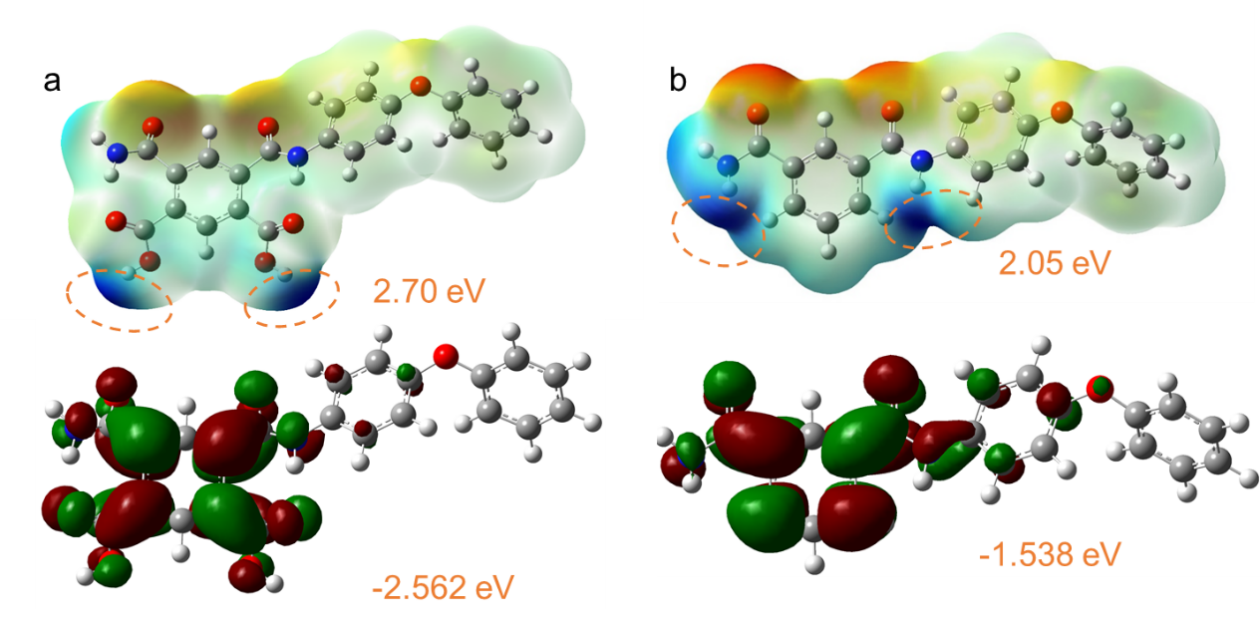


**Fig. S1** (a) Electrostatic potential (ESP) map and lowest unoccupied molecular orbital (LUMO) of PAA, a lower LUMO energy level (-2.562 eV) and a higher trap depth (2.7 eV) in the ESP map, indicating that PAA had a stronger electron-withdrawing ability. (b) Electrostatic potential (ESP) map and lowest unoccupied molecular orbital (LUMO) of PA. PA's low electron-withdrawing ability was demonstrated by its high LUMO energy level (-1.538 eV) and shallower trap depth (2.05 eV).


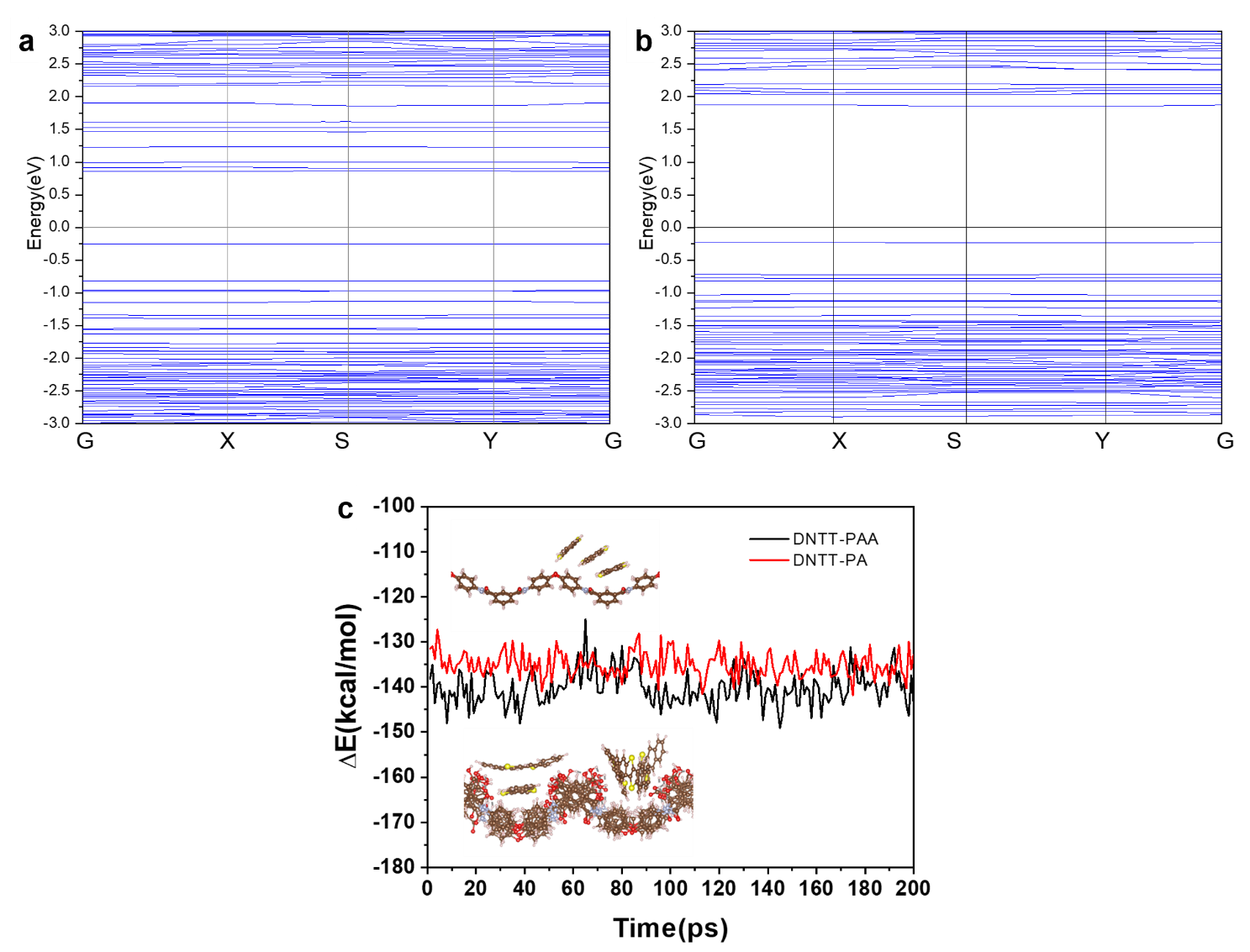


**Fig. S2** Band structures of DNTT grown on the PAA (a) and PA (b) dielectric layers. The energy band gap of PAA/DNTT system is 1.5 eV and PA/DNTT system is 2.4 eV. (c) The calculated interaction force between the DNTT and dielectrics. PAA/DNTT system has stronger interaction between the dielectric layer and the semiconductor.


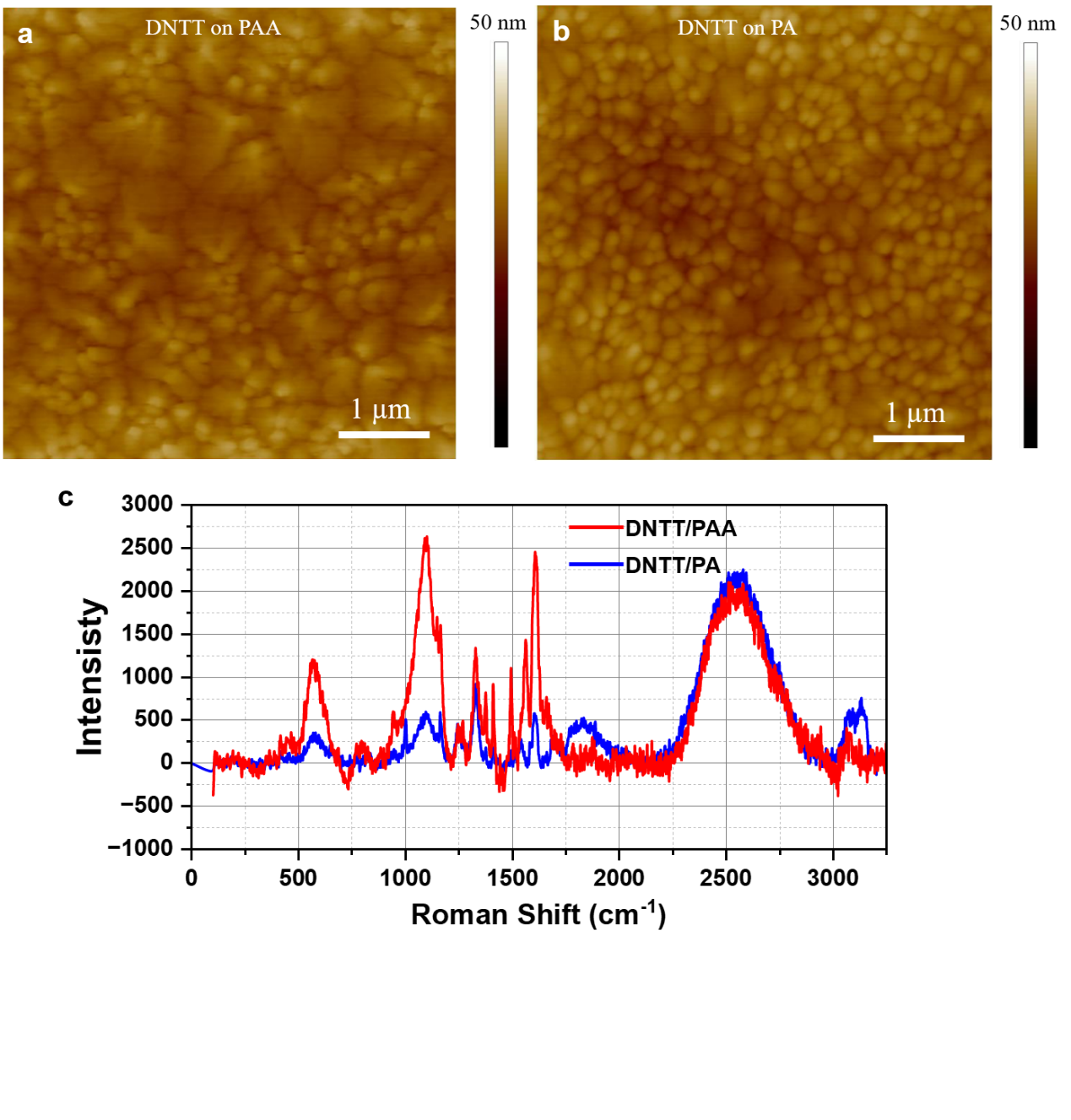


**Fig. S3** AFM images of DNTT films grown on (a) PAA and (b) PA dielectric layer. The grain size of the DNTT film on the PAA surface was significantly bigger as compared to the PA surface. (c) Raman spectrum of DNTT films on PAA and PA dielectric layers.


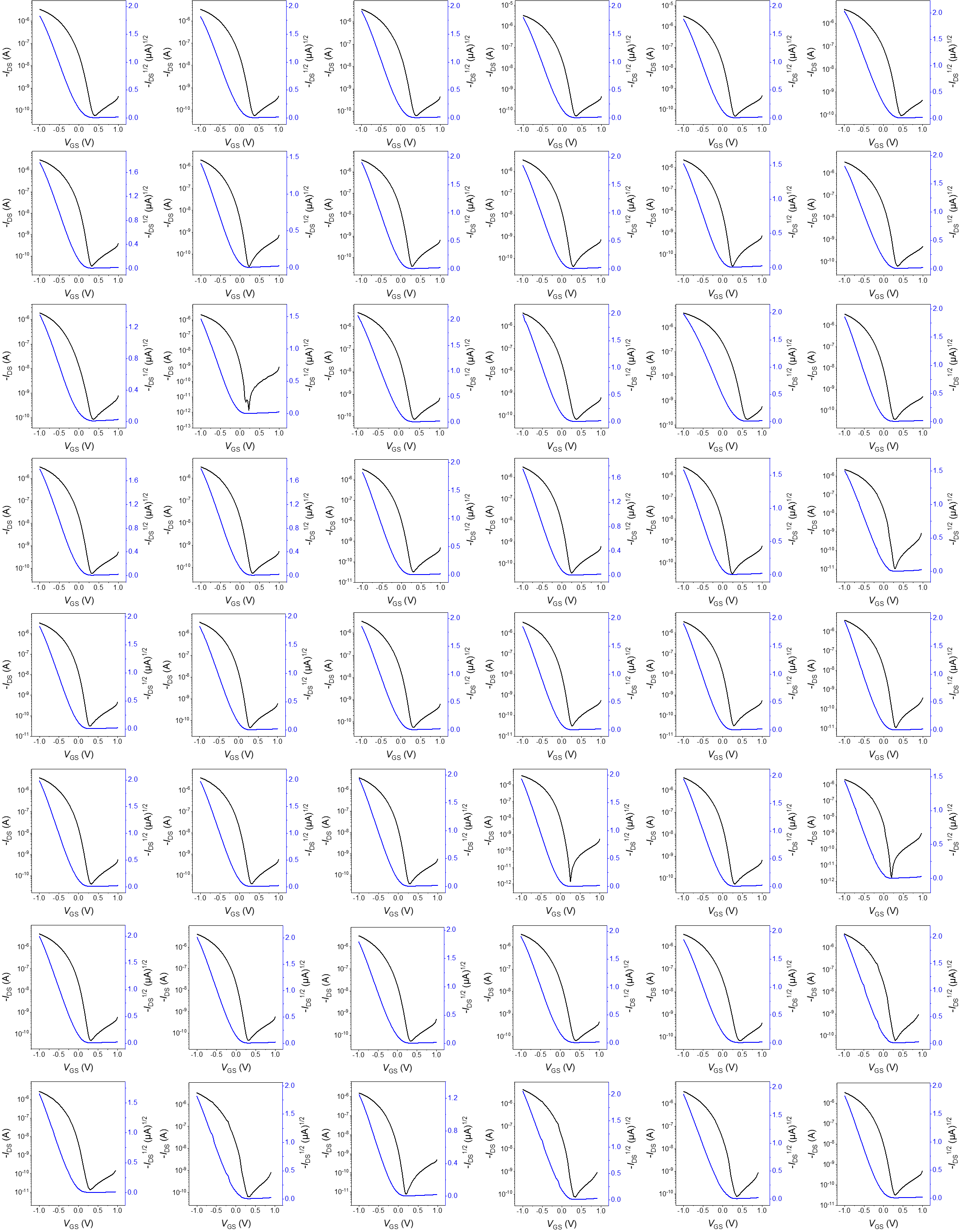
**Fig. S4** Typical transfer curves of the OFET arrays (6×8) with 20 nm DNTT as the active layer (V_D_ = -1 V) and PAA as dielectric layers.


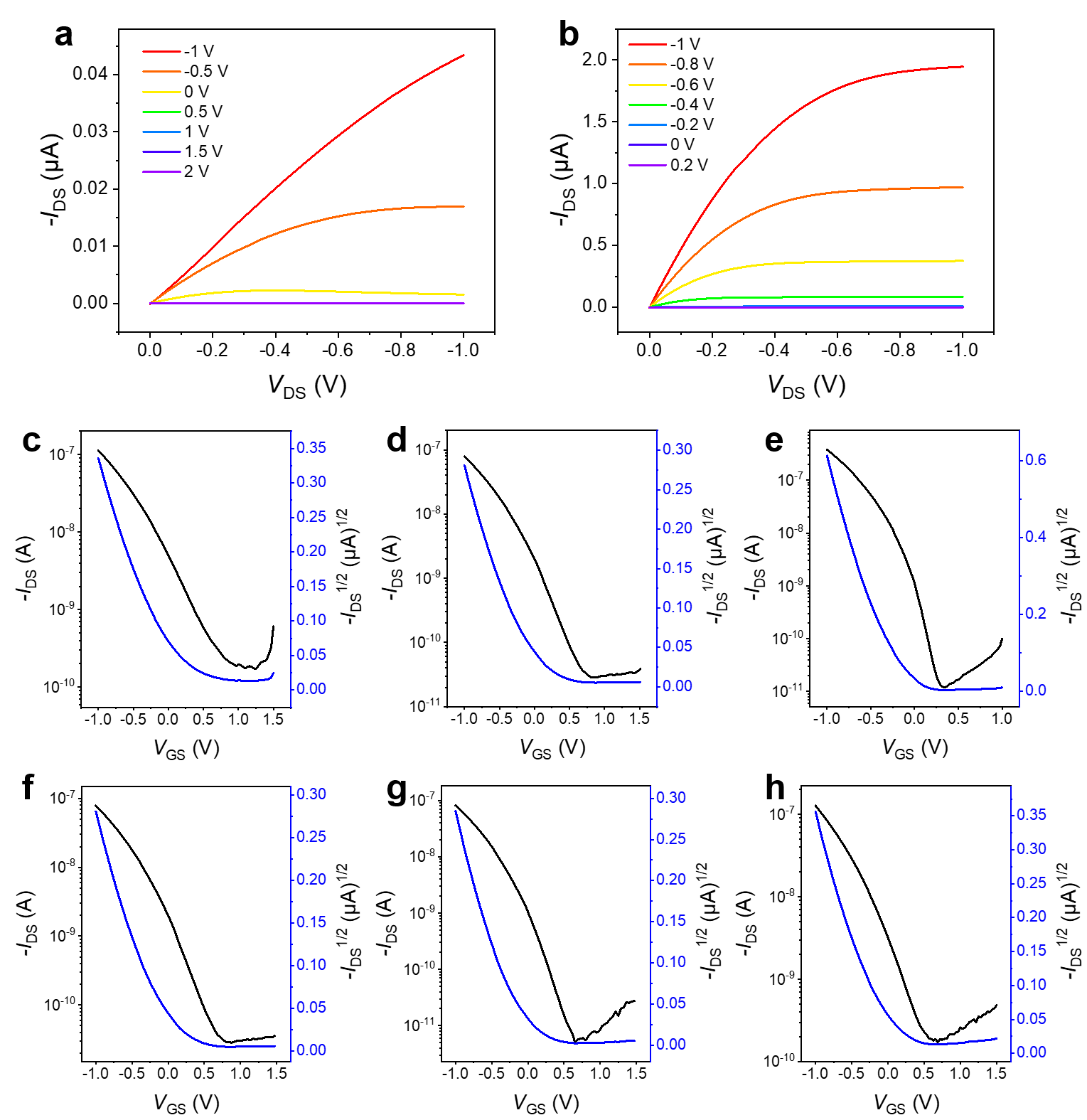


**Fig. S5** (a) Typical output curves of the OFET with PA as the dielectric layer. (b) Typical output curves of the OFET with PAA as the dielectric layer. (c-h) Typical transfer curves of the OFET with 20 nm DNTT as the active layer (V_D_ = -1 V) and PA as dielectric layers.


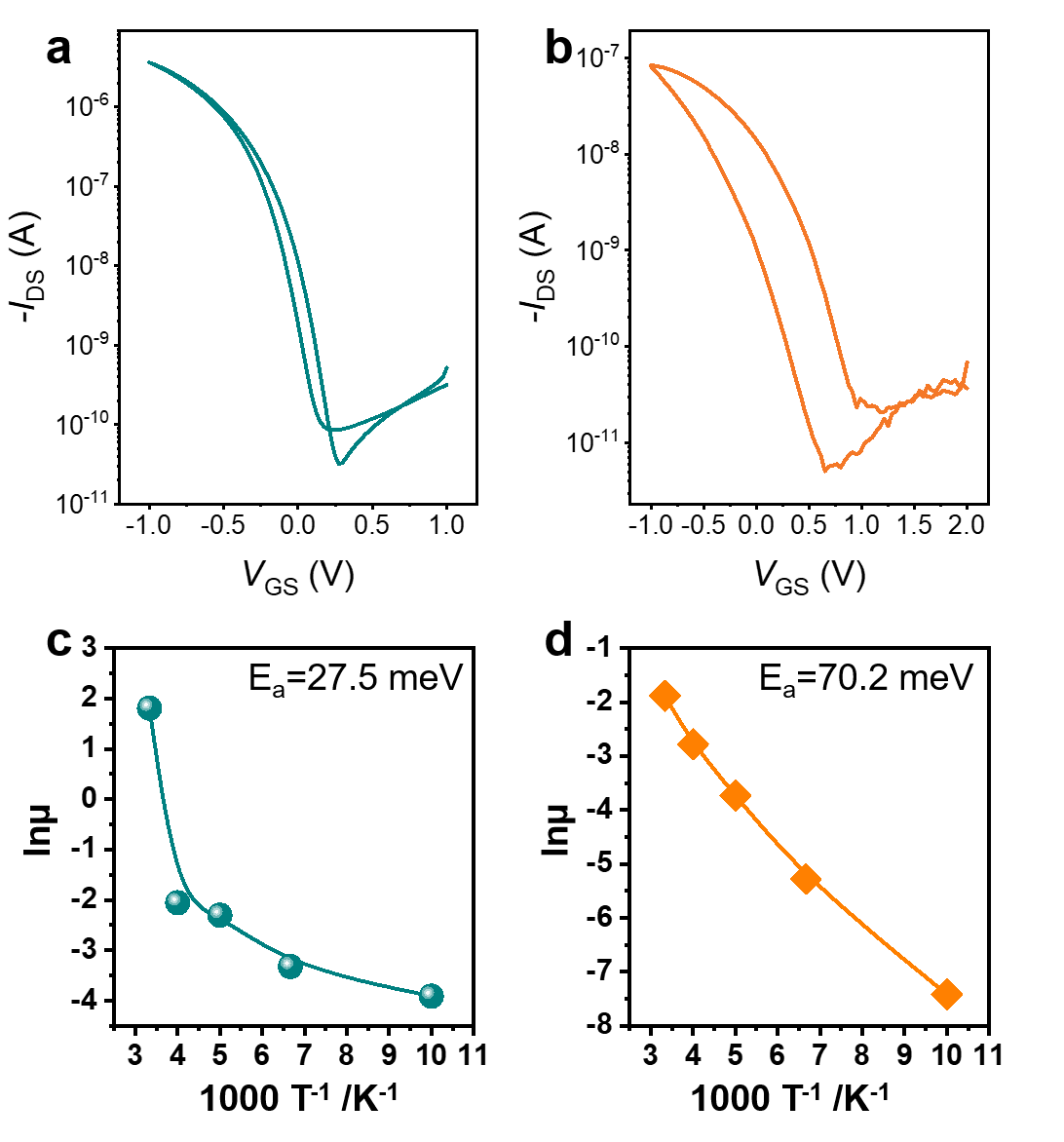


**Fig. S6** Typical transfer curve (both forward and reverse sweeps) of the device with PAA (a) and PA (b) as dielectrics. ln(μ) versus 1000/T (c) using PAA-based OFETs and (d) using PA-based OFETs.


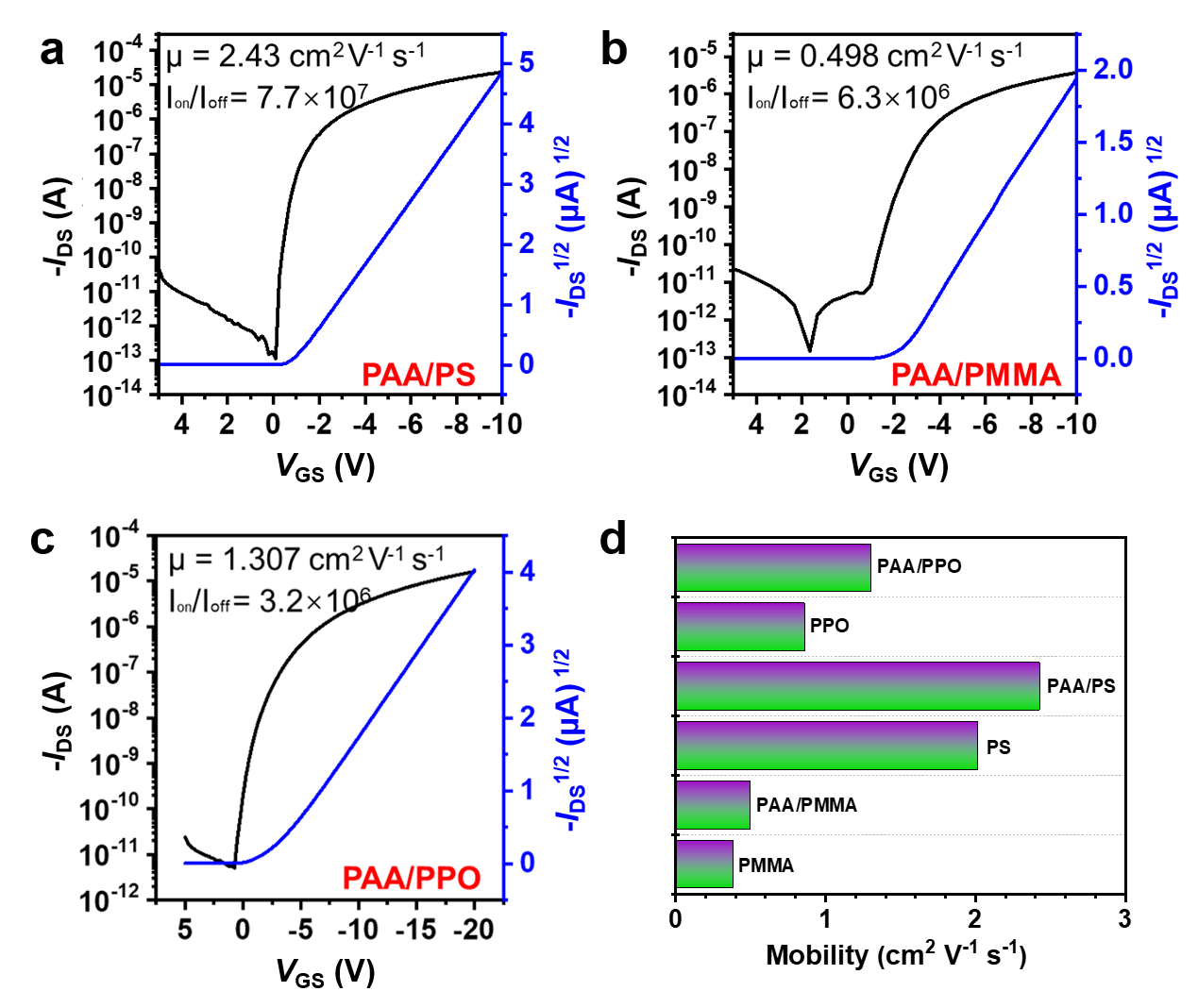


**Fig. S7** Typical transfer curves of the OFET with 20 nm DNTT and a channel dimension of W = 200 μm, L = 25 μm based on (a) PAA/PS; (b) PAA/PMMA; (c) PAA/PPO dielectric layers. (d) The mobility distribution of device with different polymer dielectrics.


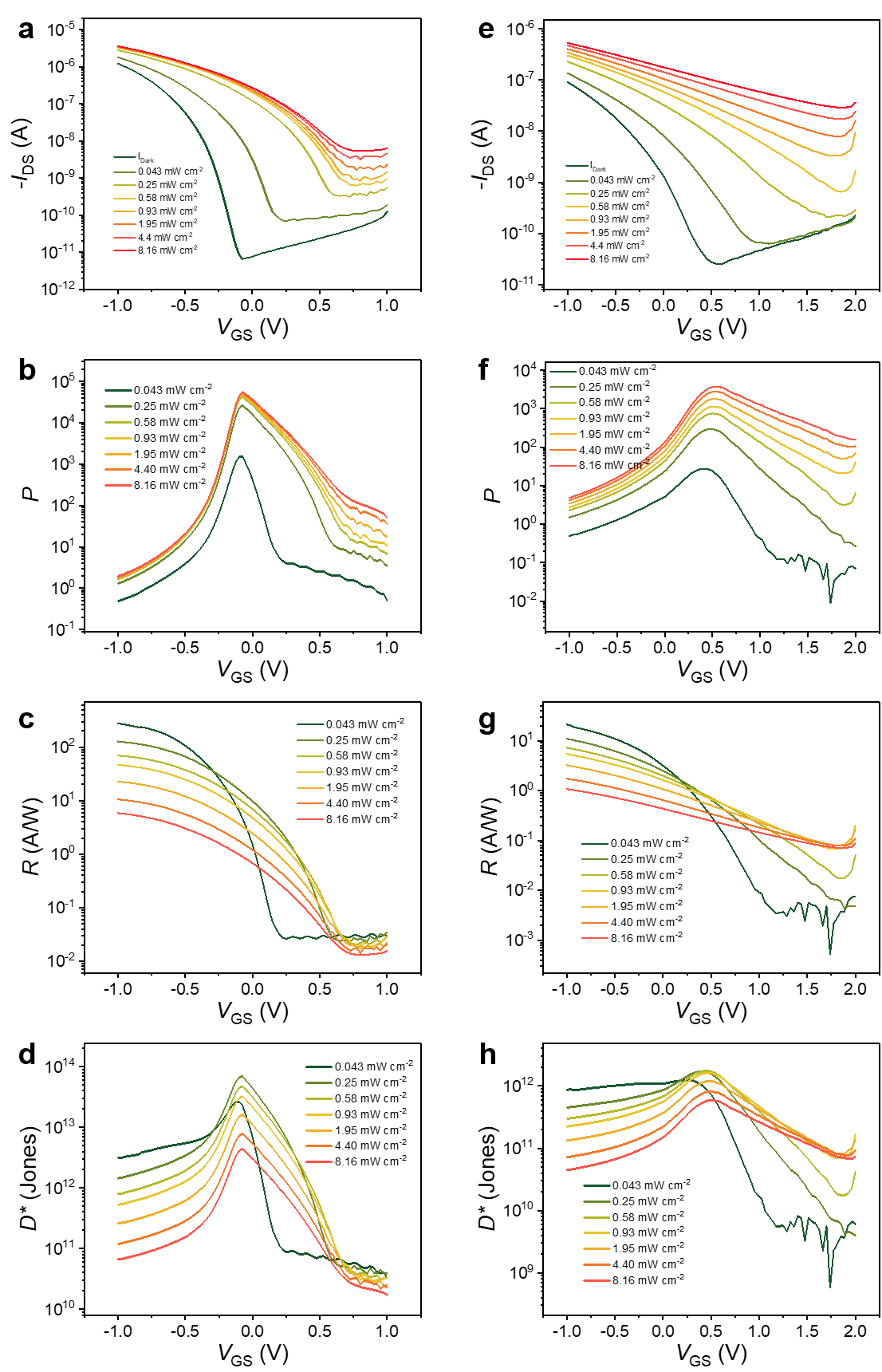


**Fig. S8** (a) Transfer characteristics of PAA-based phototransistor measured under different illumination intensities in the air. P (b), R (c) and D^*^ (d) as a function of gate voltage under differernt illumination intensity using PAA-based phototransistor. (e) Transfer characteristics of PA-based phototransistor measured under different illumination intensities in the air. P (f), R (g) and D^*^ (h) as a function of gate voltage under differernt illumination intensity using PA-based phototransistor.


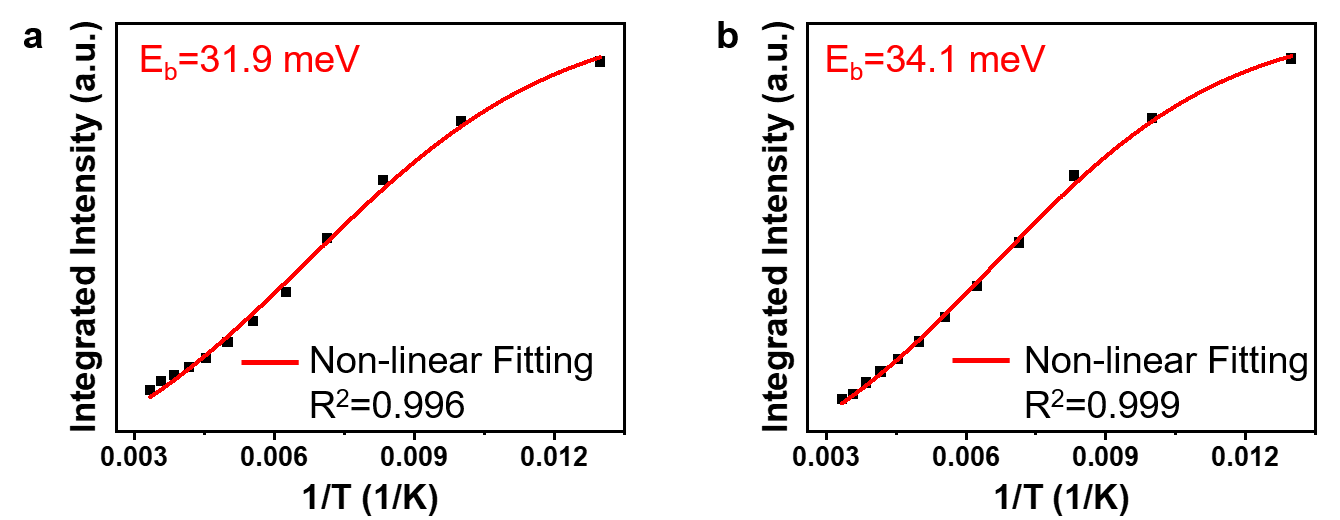


**Fig. S9** Evolution of PL intensity as a function of temperature from 77 to 300 K for DNTT/PAA (a) and DNTT/PA (b) films.


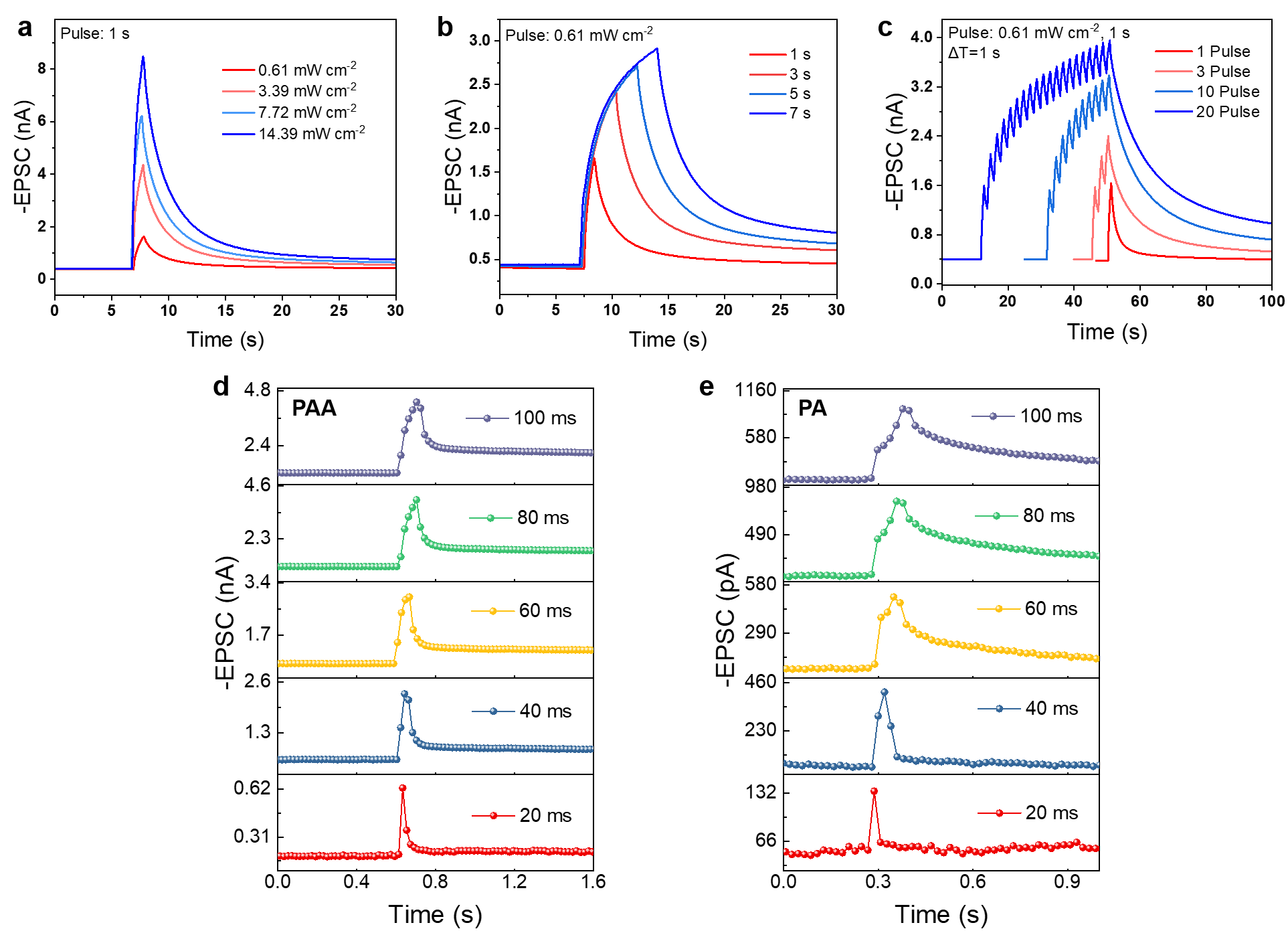


**Fig. S10** The relationship between EPSC of PA device and a) light pulse intensity, b) light pulse width, and c) light pulse number (source-drain voltage V_D_ = -0.1 V, gate voltage V_G_ = 0.12 V). EPSC of the PAA (d) and PA (e)-based devices triggered by a short light pulse of 20, 40, 60, 80, and 100 ms.


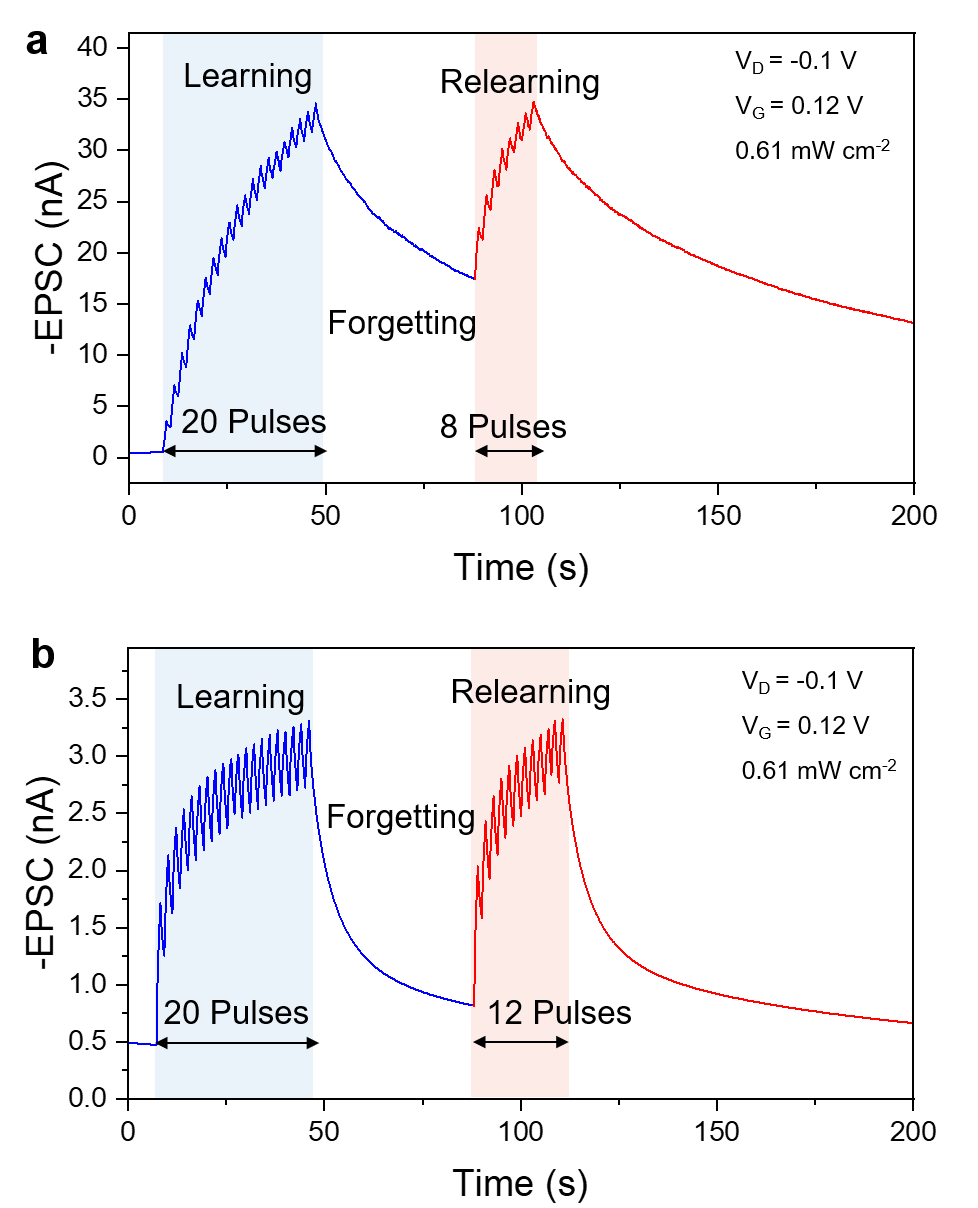


**Fig. S11** The stimulation of “learning-forgetting-relearning” process of biological synapses using the a) PAA device and b) PA device (light spike: 450 nm, 0.61 mW cm^-2^, 1 s). The first learning of both devices was carried out by applying 20 pulses which resulted in the EPSC of the PAA device increasing to 34 nA for the PAA-based device and 3.3 nA for the PA-based device within 40 s. After the same amount of forgetting, the PAA-based device's relearning process only takes 8 pulses to reach the current level of the first learning, whereas the PA device required 12 pulses to achieve the first learning effect.


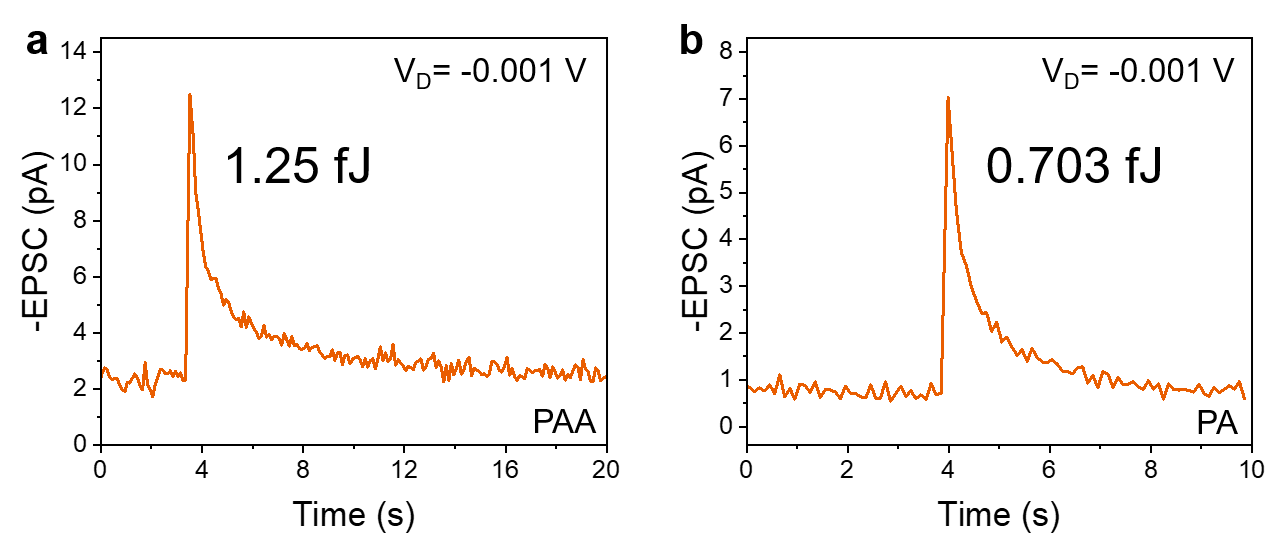


**Fig. S12** The energy consumption of the PAA device triggered by light spike (V_D_ = -0.001 V, pulse width: 0.1 s).

**Fig. S13** The frequency dependence of capacitance (PA dielectric layer).

**Table S1** List of basic parameters of OFET device based on PAA and PA dielectrics.


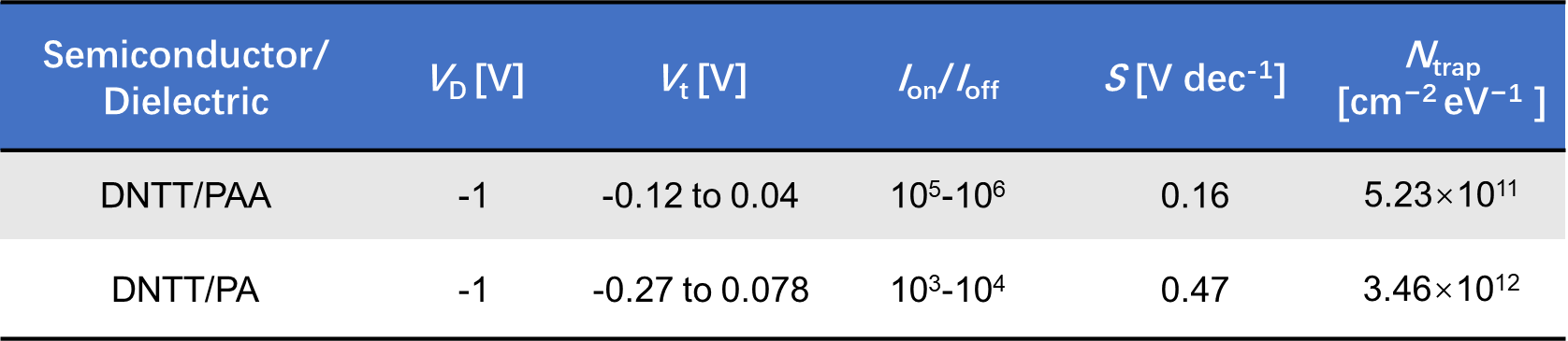


**Table S2** List of basic parameters of OFET device based on PAA and PA dielectrics using different semiconductors.


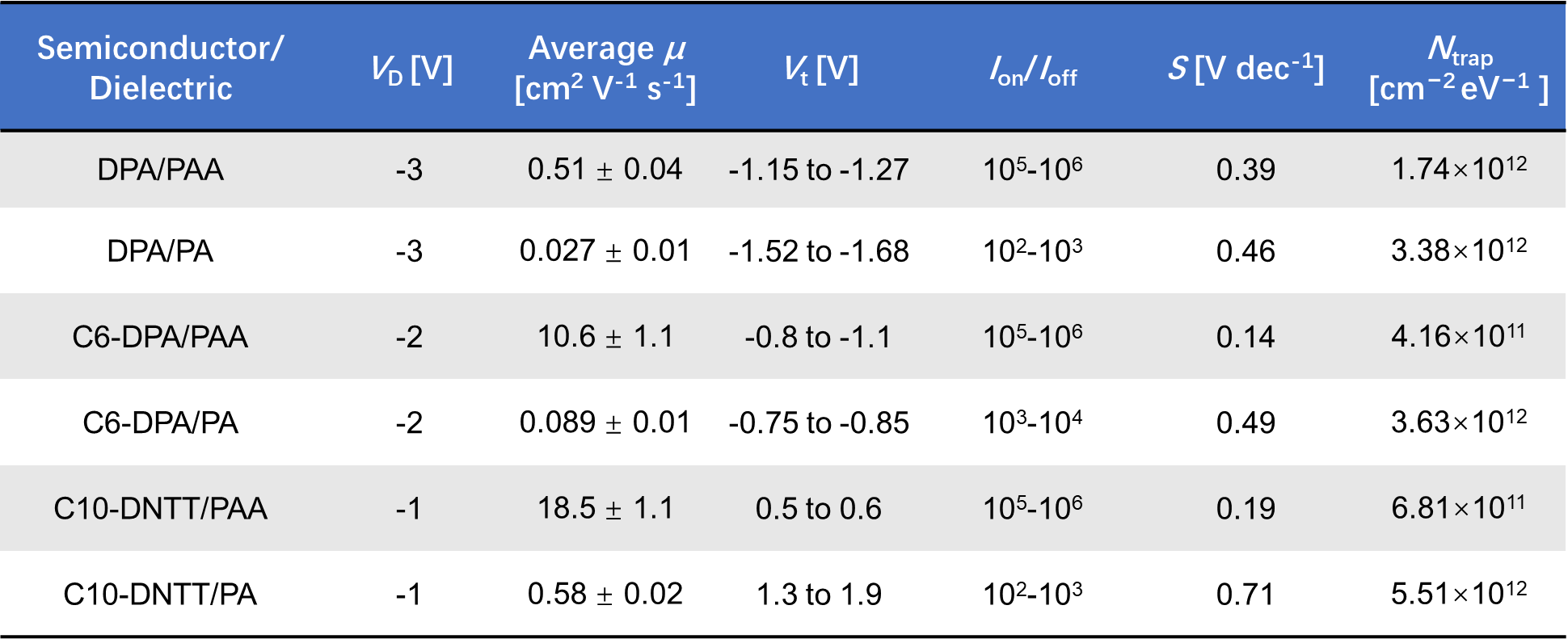

Supplement: Supplementary file 1 — Supplementary Information for Retina-inspired Organic Neuromorphic Vision Sensor with Polarity Modulation for Decoding Light Information [file 41377_2023_1310_MOESM1_ESM.docx]
